# Somatic mutations of *STK11* gene in human papillomavirus positive and negative penile cancer

**DOI:** 10.1186/1750-9378-8-2

**Published:** 2013-01-10

**Authors:** Clorinda Annunziata, Luigi Buonaguro, Simona Losito, Franco M Buonaguro, Maria Lina Tornesello

**Affiliations:** 1 Molecular Biology and Viral Oncology, National Cancer Institute "Fond. Pascale", Cappella Cangiani, 80131, Naples, Italy; 2 Department of Pathology, National Cancer Institute "Fond. Pascale", Naples, Italy

**Keywords:** HPV, STK11, Somatic mutations, Penile cancer

## Abstract

**Background:**

Human papillomavirus (HPV) infection accounts for about 40-50% of all cases of penile carcinoma suggesting that other factors, including host genetic status, are involved in neoplastic transformation. In this perspective, *STK11* gene, which has been found frequently mutated in HPV-related cervical carcinoma, has been analyzed in HPV-positive and HPV-negative invasive penile cancers to establish its mutational status and the possible correlation of HPV infection with specific genetic alterations.

**Methods:**

Genomic DNAs extracted from 26 cases of penile squamous cell carcinoma were analyzed for genetic alterations in the exons 1 to 9 of *STK11* gene by quantitative real-time PCR. Ratios of potentially deleted and non-deleted exons were indicative of specific loss of *STK11* coding regions. DNA samples of 5 cancer cases were subjected to standard PCR amplification of *STK11* exons 1 to 9 and analyzed for somatic mutations by direct nucleotide sequencing analysis.

**Results:**

Heterozygous deletions of *STK11* exon 1 and 2 were identified in 2 out of 14 HPV-positive (14.3%) and 1 out of 12 HPV-negative cases (8.3%). Complete nucleotide sequencing analysis of exons 1 to 9 showed a single nucleotide change upstream the exon 2 coding region in 1 out of 5 penile carcinoma samples.

**Conclusions:**

The present results suggest that single nucleotide mutations and/or deletions of *STK11* gene are rare events in penile cancer. Moreover, no significant association was observed between *STK11* alterations and HPV infection in these tumors.

## Background

Penile cancer is a rare malignancy in Western Europe and the United States with age-standardized incidence rates (ASR) of 0.1-1.5 per 100,000 men. In Africa, Asia and South America, however, the estimate ASRs are significantly higher with peaks of 2.8 and 3.7 per 100,000 men, in Uganda and Brazil, respectively [1].

The most common histological type of penile carcinoma is the squamous cell carcinoma (95%) which comprises several subtypes such as usual squamous cell carcinoma(48-65%), basaloid (4-10%), warty (7-10%), verrucous (3-8%), papillary (5-15%) and mixed carcinoma (9-10%) [2]. Although the etiology of penile cancer is not yet fully understood several risk factors, such as poor hygiene and phimosis, lack of circumcision in childhood and history of smoking, have been shown to increase the risk to develop this malignancy [3-6]. Human papillomaviruses (HPV) have been associated with approximately 47% of invasive penile carcinoma cases with HPV16 and HPV18 as the most common viral genotypes accounting for 60.23% and 13.35%, respectively, of the HPV attributable cases [7]. Higher rates of HPV positivity have been found in warty-basaloid (82%), basaloid (76%), and warty carcinomas (39%). This observation was indicative of a strong association between the basaloid cell type and presence of HPV [8].

Several studies have reported genetic alterations in tumor suppressor genes and oncogenes, in both HPV-positive and HPV-negative penile cancers, which may have a critical role in tumor carcinogenesis [9,10]. *STK11* is a 23 kb tumor suppressor gene, mapped to chromosome 19p13.3, which encodes for the serine/threonine kinase 11 (STK11) also known as liver kinase B1 (LKB1) or renal carcinoma antigen NY-REN-19 [11,12]. The *STK11* gene is widely expressed in embryonic and adult tissues and the encoded STK11 kinase is an essential regulator of chromatin remodeling, cell cycle arrest, p53-dependent apoptosis, Wnt signaling, cell polarity and energy metabolism [13-15]. Germ-line mutations of the *STK11* gene are associated with Peutz-Jeghers syndrome (PJ) [11,12], an autosomal dominant disorder characterized by hamartomatous polyps of the gastrointestinal tract and by a considerably increased risk of cancer in gastrointestinal tract, pancreas, breast, lung, uterus, cervix, ovary and testis [16-18]. PJ patients are at increased risk to develop cancer particularly at body sites with higher levels of STK11 enzyme in the normal tissue [19].

Somatic mutations of *STK11*gene have been frequently found in several sporadic tumors, including HPV-related cervical cancer [20-25]. No studies have been performed on *STK11* mutational status in penile carcinoma.

The aim of the present study was to analyze genetic alterations and single nucleotide mutations in exons 1 to 9 of *STK11* gene in HPV-positive and HPV-negative penile cancers to possibly establish a relationship between HPV infection and genetic alterations involved in cancer progression.

## Methods

### Samples and DNA isolation

This study included DNA samples extracted from liquid-nitrogen frozen specimen of penile squamous cell carcinoma (n = 6) from Black Ugandan patients and paraffin-embedded penile squamous cell carcinoma (n = 20) from Caucasian Italian patients. These samples were previously characterized in terms of histology, DNA quality, HPV genotypes, HPV16 variants and viral integration status [9,26]. The study protocol was approved by the ethical review board of the involved Institution. Genomic DNA was extracted from frozen biopsies as well as from thin sections of fixed and embedded tissues according to published procedures [27,28]. In particular two 10 μm sections of each biopsy were extracted twice with 1 ml of xylenes, for paraffin removal, and twice with 500 μl of 100% ethanol, for organic solvents removal. Both xylenes-treated and fresh tissue samples were digested with Proteinase K (150 μg per ml at 60°C for 30 min) in 100-500 μl lysis buffer (10 mM Tris-HCl pH 7.6, 5 mM EDTA, 150 mM NaCl, 1% SDS), followed by DNA purification by phenol and phenol-chloroform-isoamyl alcohol (25:24:1) extraction and ethanol precipitation in 0.3 M sodium acetate (pH 4.6). Genomic DNA was also extracted from PC23, HeLa and SiHa cell lines to be used as positive and negative control, respectively, in the PCR and real time PCR.

### Quantitative real-time PCR

Exons 1 to 9 of *STK11* gene were independently amplified by real time polymerase chain reaction (PCR) using primer sequences listed in Table 1. The nine primer pairs have been designed with Beacon Designer 7.9 (Bio Rad Laboratories, Inc). All *STK11* exons were amplified in a total volume of 25 μl containing iQ SYBR Green Supermix containing 50 mM KCl, 20 mM Tris-HCl, pH 8.4, 0.2 mM of each dNTP and 25 units/ml iTaq DNA polymerase, 3 mM MgCl2, 10 nM SYBR Green I (Bio-Rad Laboratories, Inc), 3 μmol/L of each primer, and 100 ng - 500 ng of genomic DNA. All experiments were performed on the CFX96 Real Time System (Bio-rad Laboratories, Inc). The specificity of amplification was confirmed applying dissociation analysis starting at 65°C.

**Table 1:** **Primer sequences used for PCR, nucleotide sequence analysis and Real-time PCR amplification of *STK11 *exons 1 to 9**

**Region**	**Forward (5’-3’)**	**Reverse (5’-3’)**	**Product length**	**Annealing temperature (°C)**
*STK11* exon 1	ACAATCGTTTCTGTTGGAAG	TCCTTGTGTTCCGACTTC	113 bp	55
*STK11* exon 2	CTCTAGGGAAGGGAGGAG	TTTCTGCTTCTCTTCGTTG	201 bp	61
*STK11* exon 3	TGAGCTGTGTGTCCTTAG	CACTGGGAAACGCTTCTC	102 bp	60
*STK11* exon 4	CCCGCAGGTACTTCTGTC	GTGGTGAGCAGCAGGTTC	100 bp	61
*STK11* exon 5	CCTGAGGGCTGCACGGCACC	GGGGCGGGGCACTTACAGG	180 bp	69
*STK11* exon 6	TCGAAATGAAGCTACAACATC	TTTCAGCAGGTCAGAGAG	138 bp	61
*STK11* exon 7	TTCTCCTCAGGGATGCTTG	CACCTGTGCTGCCGGATC	71 bp	60
*STK11* exon 8	ATGACTGTGGTGCCGTAC	CCGTGAAGTCCTGAGTGTAG	100 bp	61
*STK11* exon 9	CAGGACAGGTCCCAGAAGAG	CCAGCCTCACTGCTGCTT	203 bp	67

A 5-log dilution series of genomic DNA, extracted from the cell line PC23 [29], were amplified in each experiment for the construction of an absolute standard curve. This curve was used to determine the copy number of exon 1(test) and exon 7 (reference) of *STK11* gene in the unknown samples. All PCR reactions were set up in duplicate.

Deletions of *STK11* regions were verified by calculating the ratios (R) of potentially deleted and non-deleted exons. Values of R between 0 and 0.3 and between 0.4 and 0.7 are assumed to be indicative of homozygous or heterozygous deletions, respectively; values of R between 0.8 and 1.2 indicate no changes of the copy number in the two exons.

### Mutation analysis of *STK11* gene

Exons 1 to 9 of *STK11* gene were independently amplified by polymerase chain reaction (PCR). All primer sets, described in Table 1, were used to perform standard PCR reactions in 50 μl reaction mixture containing 100–500 ng of target DNA, Hot Master buffer containing 2.5 mM MgCl_2_, 20 pmol of each primer, 200 μM of dNTPs mix and 1.25 units of thermostable Taq DNA polymerase (5-Prime GmbH, Hamburg, DE). DNA was amplified in a Perkin-Elmer GenAmp PCR System 9700 thermal cycler with the following steps: an initial 2 min denaturation at 94°C, followed by 36 cycles of 30 s at 94°C, 30 s at the annealing temperature specified in Table. 1, 72°C for 30 s, and a final elongation at 72°C for 5 min. A reaction mixture without template DNA was included in each PCR run, as negative control. All products were analyzed by electrophoresis on 7% plyacrylamide gels to verify the specificity of the PCR product. Amplimers of *STK11* exons 1–9 from 5 samples were subjected to automated direct nucleotide sequence analysis at Primm Laboratories (Milan, IT) using the same primers used for PCR amplification. *STK11* mutations were sought by comparison analysis with the NG_007460.1 reference sequence present in the GenBank.

## Results

**Figure 1:**
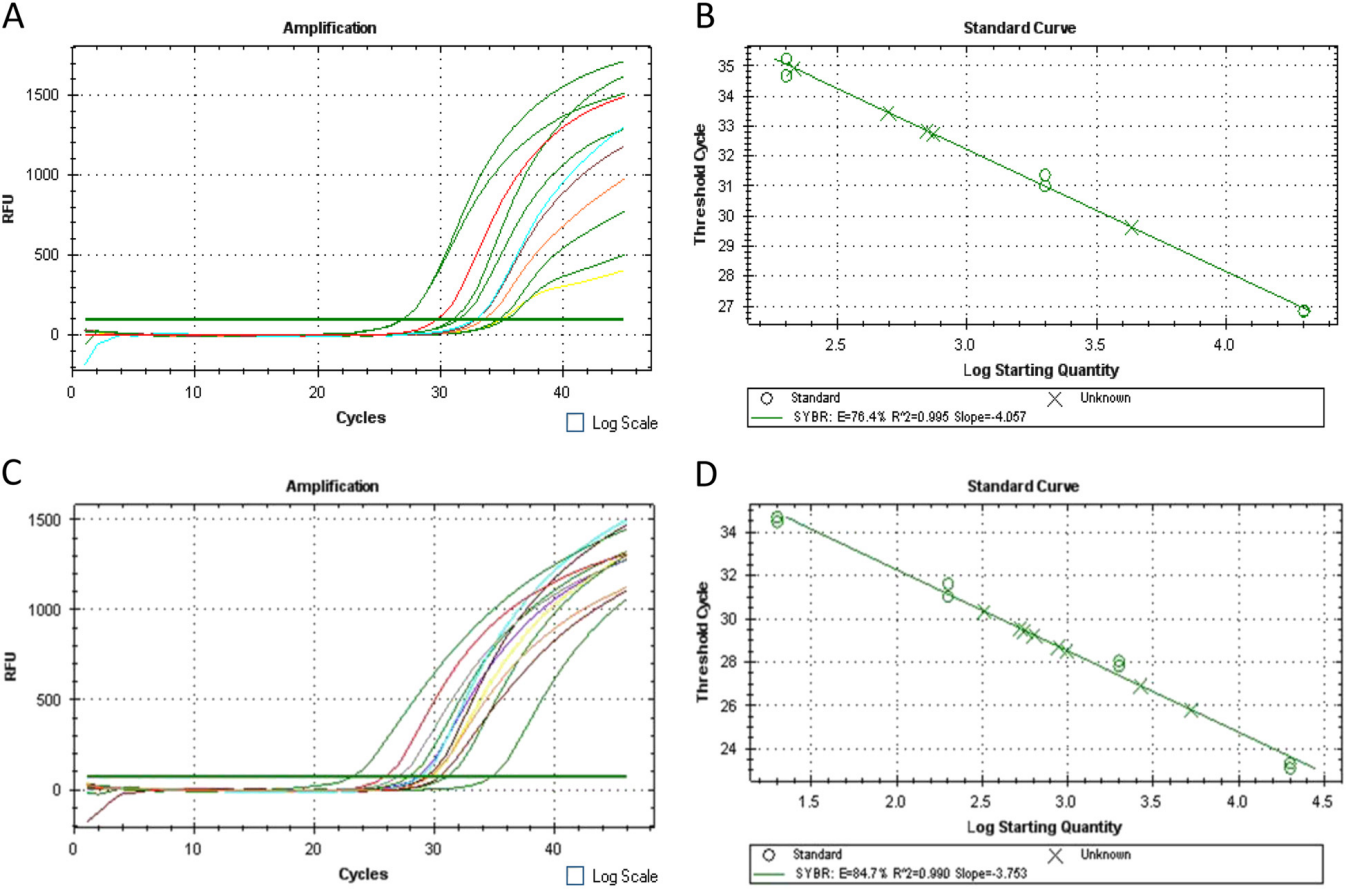
**Quantitative Real-time PCR of exon 1 and exon 7 of *STK11 *gene in penile cancers.** Amplification of exon 1 (**A**) and exon 7 (**C**) of *STK11* gene. Standard curves of 5-log dilution genomic DNA (PC23 cell line) used to determine the copy number of exon 1(**B**) and exon 7 (**D**) of *STK11* gene in the unknown samples.

Fourteen HPV-positive (53.9%) and twelve HPV-negative (46.2%) penile squamous cell carcinoma samples were analyzed for genetic alterations by real-time PCR using specific primer sets designed to individually amplify exons 1 to 9. While exons 3 to 9 were always amplified, exons 1 and 2 were not efficiently amplified in 3 out of 26 (11.5%) samples. All 26 samples were subjected to a further quantitative real-time PCR using primer pairs specific for exon 1 (test) and for exon 7 (reference) to determine an absolute quantification of genomic equivalents of exon 1 and exon 7 in each sample. A 5-log dilution series of genomic DNA, extracted from the cell line PC23, was also amplified in the same reaction set to obtain an amplification standard curve (Figure 1). As expected, in 23 out of 26 samples the ratio of exon 1/exon 7 copy number was R ≥ 0.8, indicating no deletions in exon 1. In three out of 26 cases the ratio of exon 1/exon 7 was R ≈ 0.5, indicating the presence of an heterozygous deletion in the test exon. In particular, *STK11* deletions were identified in 2 out of 14 HPV positive (14.3%) and 1 out of 12 HPV-negative cases (8.3%) (Table 2). All three *STK11* deleted cases were of Italian origin.

**Table 2:** List of penile cancer samples analysed for exon’s deletions

**Sample**	**HPV**	**Exon 1 copy number**	**Exon 7 copy number**	**Exon 1/Exon 7 ratio**	**Change of exon 1 copy number**	**Origin**
PC04	HPV16	3.0 × 10^4^	3.1 × 10^4^	1	no change	Uganda
PC07	HPV16	3.6 × 10^4^	3.5 × 10^4^	1	no change	Uganda
PC08	HPV16	2.1 × 10^3^	2.2 × 10^3^	1	no change	Uganda
PC15	HPV16	4.0 × 10^4^	4.2 × 10^4^	1	no change	Uganda
PC17	HPV16	2.8 × 10^3^	2.7 × 10^3^	1	no change	Uganda
PC23	HPV16	4.0 × 10^3^	3.8 × 10^3^	1.1	no change	Uganda
PC30	HPV16	480	457	1.1	no change	Italy
PC31	Negative	648	651	1	no change	Italy
PC32	HPV16	787	840	0.9	no change	Italy
PC33	Negative	637	765	0.8	no change	Italy
PC34	Negative	838	943	0.9	no change	Italy
PC35	HPV 18	487	847	0.5	heterozygous deletion	Italy
PC36	HPV16	950	975	1	no change	Italy
PC37	HPV16	655	645	1	no change	Italy
PC38	HPV16	780	795	1	no change	Italy
PC79	HPV16	501	495	1	no change	Italy
PC80	Negative	457	432	1.1	no change	Italy
PC81	Negative	380	347	1.1	no change	Italy
PC82	Negative	468	310	1.4	no change	Italy
PC84	Negative	930	971	1	no change	Italy
PC85	HPV56/54	172	316	0.5	heterozygous deletion	Italy
PC90	Negative	215	475	0.4	heterozygous deletion	Italy
PC91	Negative	697	846	0.8	no change	Italy
PC92	Negative	461	553	0.8	no change	Italy
PC93	Negative	738	643	1.1	no change	Italy
PC94	Negative	806	780	1	no change	Italy

**Figure 2:**
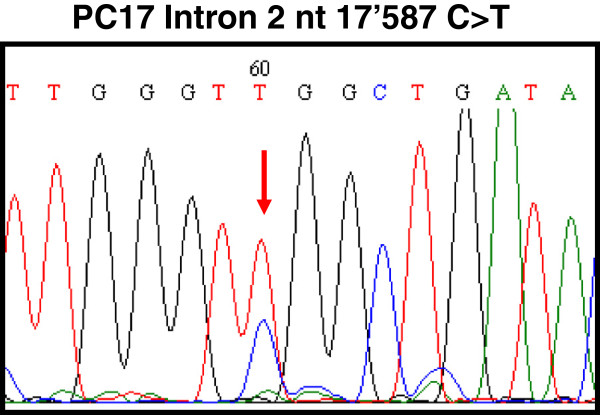
DNA sequence electropherogram showing C to T transition at nucleotide 17587 in intron 2 of STK11 gene.

Moreover, nucleotide sequencing analysis of the whole coding region (exons 1 to 9) of *STK11* gene was performed in 5 samples. A single nucleotide mutation, consisting of C to T transition at position 17587 in the intronic region 2 was detected in one case from Uganda out of 5 (20%) total samples analyzed (Figure 2).

## Discussion

The knowledge of genetic alterations in penile cancer is limited. In this study we investigated *STK11* somatic mutations in both HPV-positive and HPV-negative penile squamous cell carcinoma cases, in order to verify a potential role of this tumor suppressor gene in penile tumorigenesis. The results obtained in our study showed that *STK11* exon 1 and 2 are deleted in 11.5% of penile cancers. In particular, heterozygous deletions of *STK11* exons 1 and 2 were observed in 14.3% of HPV-positive and in 8.3% of HPV-negative tumors, indicating that HPV status has no effect on the genetic alteration of *STK11* gene.

Genetic alterations of *STK11*gene have been identified in many sporadic cancers and derived cell lines, including HPV-related cervical carcinoma. In particular, Wingo et al. showed that up to 20% of cervical cancers harbored somatic mutations in *STK11* coding regions, of which approximately one-half were single nucleotide substitutions or small deletions while the other half consisted of larger monoallelic or biallelic deletions [25]. Moreover they showed that the inactivation of this gene was associated with accelerated disease progression. Furthermore, an homozygous deletion of whole or of part of *STK11*gene was observed in SiHa and HeLa cell line [24,25].

The nucleotide sequence analysis of exons 1 to 9 of *STK11* in 5 penile carcinoma cases showed no somatic mutations in none of the 9 coding regions. A single nucleotide substitution was identified in the intron 2 of one cancer case, however it seems to have no effect on the expression of the protein. Somatic mutations, affecting conserved residues within the kinase domain or intron/exon junctions of *STK11*, were previously identified in 4 out of 6 cases (66.7%) of cervical adenocarcinomas and 2 out of 10 cases (20%) of squamous cell carcinomas, indicating that single nucleotide substitutions are frequent events in a specific histological subtype of cervical cancer [25].

Very few studies are available on somatic mutations of other genes in penile carcinoma. A recent report showed a dysregulation of phosphatidylinositol 3-kinase and Ras pathways through somatic alterations of the PIK3CA, HRAS and KRAS genes in 11 out of 28 (39%) penile cancer samples, without any statistically significant difference between HPV-positive and HPV-negative cases [30].

Other studies analyzing genetic alterations in TP53 gene of penile carcinomas reported mutations in fractions varying from 8% to 33% of the cases [31-35]. Furthermore, most of reported alterations consist in single nucleotide mutations that cause amino acid substitution in the corresponding protein.

## Conclusions

The present study has the limitation to analyze a modest sample size, but has the advantage to include cancer cases from high incidence (Uganda) and low incidence (Italy) geographic regions. The results obtained so far allow to conclude that single nucleotide mutations and/or deletion of *STK11* gene are rare events in penile carcinoma suggesting that *STK11* genetic alterations do not have a relevant role in the pathogenesis of penile cancer.

## Abbreviations

HPV: Human Papillomavirus;STK11: Serine/threonine kinase 11;LKB1: Liver kinase B1.

## Competing interests

The authors report no competing of interests.

## Authors’ contribution

MLT and FMB were responsible for the overall planning and coordination of the study. LB contributed to the data analysis. SL carried out the histopathology evaluation of the cases. CA was responsible for specimen processing, DNA analysis and with MLT compiled and finalized the manuscript. All authors read and approved the final manuscript.
